# Early-onset, conjugal, twin-discordant, and clusters of sporadic ALS: Pathway to discovery of etiology *via* lifetime exposome research

**DOI:** 10.3389/fnins.2023.1005096

**Published:** 2023-02-13

**Authors:** Peter S. Spencer, Valerie S. Palmer, Glen E. Kisby, Emmeline Lagrange, B. Zane Horowitz, Raquel Valdes Angues, Jacques Reis, Jean-Paul Vernoux, Cédric Raoul, William Camu

**Affiliations:** ^1^Department of Neurology, School of Medicine, Oregon Health and Science University, Portland, OR, United States; ^2^Oregon Institute of Occupational Health Sciences, Oregon Health and Science University, Portland, OR, United States; ^3^College of Osteopathic Medicine of the Pacific Northwest, Western University of Health Sciences, Lebanon, OR, United States; ^4^Department of Neurology, Reference Center of Neuromuscular Disease and ALS Consultations, Grenoble University Hospital, Grenoble, France; ^5^Department of Emergency Medicine, Oregon-Alaska Poison Center, Oregon Health and Science University, Portland, OR, United States; ^6^University of Strasbourg, Faculté de Médecine, Strasbourg, France; ^7^Normandie Université, UNICAEN, Unité de Recherche Aliments Bioprocédés Toxicologie Environnements, Caen, France; ^8^INM, University of Montpellier, INSERM, Montpellier, France; ^9^ALS Reference Center, Montpellier University Hospital and University of Montpellier, INSERM, Montpellier, France

**Keywords:** genotoxin, gyromitrin, agaritine, monomethylhydrazine (MMH), methylazoxymethanol (MAM), lifetime exposome, motor neuron disease

## Abstract

The identity and role of environmental factors in the etiology of sporadic amyotrophic lateral sclerosis (sALS) is poorly understood outside of three former high-incidence foci of Western Pacific ALS and a hotspot of sALS in the French Alps. In both instances, there is a strong association with exposure to DNA-damaging (genotoxic) chemicals years or decades prior to clinical onset of motor neuron disease. In light of this recent understanding, we discuss published geographic clusters of ALS, conjugal cases, single-affected twins, and young-onset cases in relation to their demographic, geographic and environmental associations but also whether, in theory, there was the possibility of exposure to genotoxic chemicals of natural or synthetic origin. Special opportunities to test for such exposures in sALS exist in southeast France, northwest Italy, Finland, the U.S. East North Central States, and in the U.S. Air Force and Space Force. Given the degree and timing of exposure to an environmental trigger of ALS may be related to the age at which the disease is expressed, research should focus on the lifetime exposome (from conception to clinical onset) of young sALS cases. Multidisciplinary research of this type may lead to the identification of ALS causation, mechanism, and primary prevention, as well as to early detection of impending ALS and pre-clinical treatment to slow development of this fatal neurological disease.

## Introduction

Amyotrophic lateral sclerosis (ALS) has an average clinical onset age of 58–60 years, an annual incidence rate of 1–2.6 cases/100,000 persons and a prevalence of ∼6 cases/100,000 (Talbott et al., 2016), with higher rates among males (GBD 2016 Motor Neuron Disease Collaborators, 2018). Inherited forms of the disease account for 5–10% of cases in the U.S., whereas the balance has no clearly defined causation (Mehta et al., 2018). While almost 50 ALS gene mutants are recognized (Smukowski et al., 2022), with many more suspected, the vast majority of ALS cases occurs sporadically. Young-onset ALS refers to patients with initial symptom onset before age 45, the percentage of which has declined markedly since 1850, with a greater proportion on the continents of Africa, Asia, and South America perhaps related to nutritional and occupational factors (Gouveia and de Carvalho, 2007; Turner et al., 2012). The etiology of sporadic ALS (sALS) is often attributed with little evidence to undefined environmental agents acting on a genetic susceptibility to the disease (Al-Chalabi et al., 2010; Vasta et al., 2021; Goutman et al., 2022). Viruses, fungi, cyanobacteria, heavy metals, pesticides, persistent toxicants, solvents, electromagnetic radiation, electric shock, cigarette smoke, DNA damage, impaired DNA repair, epigenetic changes, immune dysfunction, endocrine abnormalities, excessive exercise, professional soccer, and trauma, have all been associated with sALS (Tandan and Bradley, 1985; Chiò et al., 2005; Armon, 2009; Pritchard and Silk, 2014; Alonso et al., 2015; Coppedè and Migliore, 2015; Harwood et al., 2016; Schwartz and Klug, 2016; Riancho et al., 2018; French et al., 2019a; Filippini et al., 2020; Andrew et al., 2022; Re et al., 2022), but convincing evidence of a causative role for any of these factors has yet to be demonstrated. Among heavy metals, lead has been proposed as a risk factor for ALS (Kamel et al., 2002; Fang et al., 2010; Meng et al., 2020; Andrew et al., 2022) but the association is considered weak (Wang et al., 2014; Newell et al., 2021). One group has proposed sALS arises from an opportunistic fungal infection (French et al., 2019a,b), and another has published evidence of mixed fungal and bacterial infection in the CNS of a small number of sALS cases (Alonso et al., 2017, 2019).

While mutations are suspected and often found in familial ALS, the operation of culpable trans-generational environmental exposures cannot be excluded (Johansen et al., 2021). For example, in the case of the former ultra-high incidence of familial and sporadic ALS among the Chamorro people of Guam, where the disorder was first thought to result from a dominantly inherited genetic trait (Plato et al., 1969), disease rates fell steadily in the second half of the twentieth century such that the high incidence had essentially disappeared (Garruto et al., 1985; Chen, 1995). A comparable reduction of high-incidence ALS has occurred in the Kii Peninsula of Honshu Island, Japan, and in Papua, Indonesia, in the western half of the island of New Guinea (Kihira et al., 2005; Spencer et al., 2005; Okumiya et al., 2014). Given the disparate genetic origins of Chamorro, Japanese, and Papuan New Guinean people, coupled with the absence of any consistent mutant genotype associated with these disappearing hyperendemic foci of ALS, the etiology of Western Pacific ALS appears to be dominated by environmental factors, particularly the gradual loss of traditional food and medicinal practices that accompanied societal modernization (Spencer et al., 2020). The only known exogenous factor of consequence arising from traditional practices is to the neurotoxic seed of cycad plants (*Cycas* spp.), which were formerly used for food (Guam) or medicine (Guam, Kii-Japan and Papua-Indonesia), as recorded on film^1^. These practices resulted in systemic exposure principally to methylazoxymethanol (MAM, the aglycone of cycasin and neocycasins) and also to beta-*N*-methylamino-L-alanine (L-BMAA), compounds with genotoxic, epigenotoxic and other toxic actions on the developing and adult mammalian nervous system (Spencer et al., 2020). Guam ALS was significantly correlated with the concentration of cycasin—but not with L-BMAA—in cycad flour used for food by Chamorros (Román, 1996). A diet of Chamorro-prepared cycad flour induced unilateral arm weakness and neuropathological changes thought to be reminiscent of ALS (Dastur, 1964), mice fed washed cycad pellets developed an ALS-like syndrome, with loss of motor neurons and later loss of dopaminergic innervation of the striatum (Wilson et al., 2002), and cycasin is known to be responsible for induction of hindlimb stiffness and weakness, muscle wasting, and spinal lesions in cattle and goats grazing on cycad leaves or seed (Shimizu et al., 1986; Spencer et al., 2022b). Formaldehyde is a metabolite of both L-BMAA and MAM (Spencer et al., 2020), variably identified as a risk factor for ALS (Weisskopf et al., 2009; Pinkerton et al., 2013; Seals et al., 2017) and the potential role of formaldehyde in relation to neuronal DNA damage and ALS has been discussed (Spencer, 2018).

Beyond the Western Pacific, L-BMAA and formaldehyde have been reported to be highly associated with sALS (French et al., 2019b). L-BMAA is produced by prokaryotic (cyanobacteria) and eukaryotic (diatoms and dinoflagellates) microorganisms across the globe (Delzor et al., 2014; Nunes-Costa et al., 2020). However, L-BMAA was excluded as a risk factor in a hotspot of sALS in the French Alps, where an association was made with consumption of poisonous wild *Gyromitra* mushrooms (Lagrange et al., 2021b). Species of these fungi contain hydrazones that are metabolized to monomethylhydrazine (MMH), a genotoxic that produces a pattern of DNA damage comparable to that of MAM. Hydrazine-related chemicals occur worldwide as natural products of certain bacteria, fungi, plants, and marine organisms; in synthetic form, they are used to produce certain pharmaceuticals and agrochemicals; in the manufacture of paints, inks, and organic dyes; in the preparation of polyurethane coatings and adhesives; as corrosion inhibitors in water treatment; to remove solids in steam generators; as oxygen scavengers; as reducing agents for metal recovery, as propellants for jet aircraft, rockets and spacecraft, and, formerly (1950s–70s) for artillery (World Health Organization, 1987; Knapton and Stobie, 1993; ATSDR, 1997; European Chemical Agency on request of the European Commission, 2011; Nguyen et al., 2021; Spencer and Kisby, 2021). Given the recently discovered association between food-derived MMH and sALS in France (Lagrange and Vernoux, 2020; Lagrange et al., 2021b), further research on the possible etiologic relationship between hydrazine-related chemicals and sALS is merited.

This paper proposes a strategy to maximize the possibility of discovering exogenous factors potentially underpinning the etiology of sALS. Once these factors have been identified, prevention can follow, either in primary form (avoidance of exposure) or, in theory, secondarily *via* post-exposure therapeutic blockade of neuropathogenesis. Two principles underlie the proposed research approach: (a) focus on conjugal, single-affected twin, young-onset, migrant, and geographically clustered sALS cases and (b) lifetime search for atypical exposures to chemical agents of all types (exposome), especially those relevant to the nervous system (neural exposome) and, in particular, to substances chemically related to those implicated in Western Pacific ALS. The present paper addresses the first point and asks whether, among published ALS cases, there are plausible potential links between sALS and a history of exposure to the many sources of hydrazine-related chemicals.

## Materials and methods

Literature citations were retrieved from online databases (principally PubMed Central^®^) using Boolean search procedures. Search terms included: ALS, Lou-Gehrig disease; motor neuron(e) disease; epidemiology, clusters, cases, and studies of neurodegenerative disease; intoxication/poisoning linked to hydrazine; hydrazine-related chemicals; cycads; mushrooms/fungi; gyromitrin; pesticides; agrochemicals; organic solvents; jet airplane fumes; ALS-related regional geographic information, populations, customs, practices, military service, and war. Focus was limited to conjugal, single-affected twin, young-onset, migrant, and clustered sALS cases, and their reported and potential association with chemicals of natural and synthetic origin.

## Results

### Environmental etiology: Migrant ALS cases

Western Pacific ALS declined after WWII in all three affected populations (Spencer et al., 2020). Best studied in Guam, mean ALS incidence among males (65/100,000) and females (35/100,000) peaked in 1950–54 and then began to decline steadily through the end of the twentieth century when high-incidence motor neuron disease had disappeared (Plato et al., 2003). While the motor neuron was clinically typical of ALS elsewhere, the disorder was sometimes associated with parkinsonism and dementia in more elderly subjects, mean incidence rates of which peaked in males in 1960–64 (50/100,00) and females in 1970–74 (25/100,000), before declining in concert with ALS (Plato et al., 2003). Signs of parkinsonism have been reported in 30% a of population-based cohort of ALS patients (Calvo et al., 2019), and dementia is commonly associated with ALS, particularly in patients with a hexanucleotide repeat expansion (*C9ORF72)* in the non-coding region of chromosome 9 open reading frame 72 (Abramzon et al., 2020).

An important feature of the Western Pacific ALS Parkinsonism-Dementia Complex was the acquisition of neurodegenerative disease by persons who had migrated to, and resided for decades within, communities with a high incidence of the disease (Garruto et al., 1981; Kokubo et al., 2022). Also noteworthy was the development of ALS in persons years or decades after migration as children or teenagers from a hotspot of the Western Pacific disease (Reed and Brody, 1975; Garruto et al., 1980; Yoshida et al., 1998; Tsunoda et al., 2017). Taken together with the steady progressive decline (from 1950s to 2000) and virtual disappearance of ALS on Guam (Garruto et al., 1985; Chen, 1995) and in the two other high-incidence disease foci (Kihira et al., 2005; Spencer et al., 2005; Kuzuhara, 2007; Doi et al., 2010; Okumiya et al., 2014), these epidemiological observations provided powerful evidence in support of an environmental etiology of motor neuron disease; furthermore, since the ALS focus among former hunter-gatherers in western New Guinea was present before the introduction of any human-made chemicals, a natural causal agent seemed likely (Gajdusek and Salazar, 1982) and was subsequently linked to the medicinal use of the toxic pulp of cycad seed (Spencer et al., 1987b,^1991^). The progressive decline of the traditional use of cycad seed for food and oral medicine was also, respectively, associated with the disappearance of high-incidence ALS on Guam and in the Kii Peninsula of Honshu, Japan (Spencer et al., 1987a,^2020^).

### Young-onset sALS

#### Western Pacific ALS cases

While Western Pacific ALS was clinically indistinguishable from ALS elsewhere, it is considered part of an ALS/Parkinsonism-Dementia Complex (ALS/PDC). ALS affected young subjects (20 years and older), while atypical parkinsonism with dementia (P-D), or dementia alone, affected older persons, with all such phenotypes in at least one instance occurring in the same Guam family, while some other cases exhibited mixed forms of the neurodegenerative disease complex (Spencer et al., 2020). While unproven, it seems plausible that persons with larger exposures to the culpable environmental agent(s) developed motorsystem disease at an early age (expressed clinically as ALS) while lesser-exposed subjects who survived fatal motor neuron loss developed mixed forms of ALS/PDC expressed clinically later in life. Additionally, on Guam in particular, many with and without ALS/PDC had a stationary pigmentary retinopathy (Campbell et al., 1993), which was replicated in laboratory species treated with cycasin or MAM at an age equivalent to the second trimester of human pregnancy (Spencer, 2020; Kisby and Spencer, 2021). While the exposure age for acquisition of neurodegenerative disease in later life is unknown, subjects who moved from the high-incidence focus in Kii-Japan developed motor neuron disease 1–7 decades later (Yoshida et al., 1998; Tsunoda et al., 2017), while exposure to Guam during adolescence/young adulthood, but not childhood, correlated strongly with ALS/PDC (Borenstein et al., 2007).

#### U.S. Veterans

In 1999, ALS cases were described among young American servicemen in an age group in which the disease is usually rare. A 2003 paper reported a nationwide epidemiologic case ascertainment study of ALS occurrence during the 10-year period since August 1990 among U.S. military subjects who served in the Gulf War (August 2, 1990, through July 31, 1991) during which the U.S. and its allies fought Iraq. A significant elevated risk of ALS was found among all U.S. personnel deployed to the Gulf region and was especially high among deployed Air Force personnel (Horner et al., 2003). During 8 post-War years, 17 of 20 Gulf War Veterans (GWV) were diagnosed with ALS before age 45 years (Haley, 2003). Although a 2005 study found an excess risk for ALS generally associated with U.S. military service (Weisskopf et al., 2005), the excess risk of ALS among 1991 GWVs was limited to the decade following the War (Horner et al., 2008). Whereas ALS prevalence among GWV was 5.8 per 100,000 over 10 years after the Gulf War, there was a significantly higher prevalence (19.7 per 100,000 persons over 14 years) among a somewhat older group of U.S. Veterans deployed in support of post-9/11 conflicts (Sagiraju et al., 2020). ALS prevalence (33/2/100,000) was significantly higher in Air Force personnel relative to that of other service branches, and among tactical operation officers in comparison with general and administrative officers. Tactical operations officers consisted primarily of pilots, aircraft crew, missile, and combat operations staff. These data suggest that environmental concerns should be explored among those who routinely work with jet aircraft and develop ALS at a young age (Hayes et al., 2021; Andrew et al., 2022).

Interview of 82 mostly young (<45 years old) Gulf-deployed and non-Gulf-deployed U.S. Veterans with ALS diagnosed between 1998 and 1999 revealed familial cases in 10% and one deployed and 2 non-deployed subjects with prior service on Guam, plus a Guam-born Chamorro (Palmer and Spencer, 2002). Principal findings are shown in Box 1. Ten percent of deployed GWVs in this study were fighter pilots or involved in aircraft maintenance.

**Box 1. ALS among U.S. Gulf War Veterans.**

Principal findings of a preliminary 2002 investigation of exposures of U.S. Gulf War Veterans (GWV, *n* = 33 males) who were deployed to S.W. Asia during the period August 1, 1990–July 31, 1991) and U.S. Gulf-era Veterans (GEV, *n* = 49 males) not deployed to S.W. Asia, who were diagnosed with definite ALS (D-ALS) or probable ALS (P-ALS) between 1991 and 2001 (Palmer and Spencer, 2002). (Supported by Department of Veterans Affairs Cooperative Studies Program #500). Report available on request from Corresponding Author.

• Approximately 10% of ALS cases were familial, three others had seen service on Guam and one was Guam-born.

• Similar numbers of D-ALS and P-ALS among GWV and GEV serving in the Army, Navy or Air Force.

• Similar dates of diagnosis for D-ALS and P-ALS, with peak incidence in 1988–99 for both groups.

• One third of D-ALS and P-ALS involved with aircraft, land-based vehicles or construction.

• Chemical exposures/activities included pesticides, organic solvents, jet fuel, welding, soldering, others.

• Voluntary skin applications included a shampoo containing neurotoxic zinc pyridinethione.

• Physical exposures included radiation (radar, microwave), electromagnetic fields, electric shocks.

• Biological exposures included vaccines, sand flies, mosquitoes; several cases of prior Lyme disease.

• Physical exposures included survival training in tropics, long-distance running and physical exhaustion.

• Oral exposures included contaminated/malodorous water and large quantities of diet and other sodas.

• One familial case collected and ate mushrooms like his uncle: both developed ALS at 37–38; years of age.

• Conjugal pair, electric mechanic/industrial cleaner and wife exposed to his chemical-saturated clothing.

One Gulf-deployed Veteran not included in this preliminary study was a 32-year-old American F16 fighter pilot who flew 44 combat missions in Gulf War Operation Desert Storm, was diagnosed with ALS at age 37, and died 9 years later (Miller, 2022; Netter and Taylor, 2005). During Desert Storm operations, the U.S. Air Force relied heavily on the F16 Fighting Falcon, a multirole jet fighter uniquely equipped with an Emergency Power Unit (EPU) powered by the monopropellant H-70, which contains 70% hydrazine (N_2_H_4_) and 30% water by weight (Anon, 2022s). With the exception of 12 F16 fighter jets used by the Royal Bahrani Air Force, none of the Allied Countries (Canada, France, Italy, Kuwait, Qatar, Saudi Arabia, U.K., United Arab Emirates) used this jet fighter during Operation Desert Storm (Anon, 2022m). Iraq had experimented with hydrazine rocket fuels, including unsymmetrical dimethylhydrazine, but the U.S. Department of Defense concluded these fuels were not used by Iraq during the Gulf War. Thus, while it appears unlikely that non-U.S. Coalition service members were exposed to hydrazine rocket fuels during the 1991 Gulf War (Brown, 2006), prior investigations of toxic exposures among U.S. GWVs have failed to recognize the potential for exposures to hydrazine associated with powering up or servicing the F16 EPU (Suggs et al., 1979; Anon, 2022h; Cenciotti, 2022). Such potential exposures from F16 EPUs continue to this day; 4,600 F16s had been built by 2012, while improved versions will continue to be constructed for export customers (Bahrain, Slovakia, Bulgaria, Taiwan, Morocco, and Jordan) through 2026 (Anon, 2022k). Small numbers of F-16 variants are used for non-flying ground instruction of maintenance personnel (Anon, 2022k). Hydrazine is also used to fuel an auxiliary power unit on the Eurofighter Typhoon, but use of hydrazine in the European Union beyond 2025 is predicted to be banned (Anosseir, 2021).

### ALS clusters and hotspots

Studies on the incidence of ALS and the identification of geographical clusters have taken place in many Western countries, including the USA (Taylor and Davis, 1989; Sienko et al., 1990; Turabelidze et al., 2008; Caller et al., 2009; Reddy, 2020), Italy (Giagheddu et al., 1983, 1993; Grainieri et al., 1989; Uccelli et al., 2007), Sweden (Gunnarsson et al., 1996), Finland (Sabel et al., 2003), Denmark (Johansen et al., 2021), United Kingdom (Mitchell et al., 1998; Scott et al., 2009), Greece (Kalfakis et al., 1991), and France (Boumédiène et al., 2011; Lagrange et al., 2021b). While an aggregation of patients in a given geographic area theoretically may occur by chance or be the consequence of a statistical bias in the process of patient selection (Malaspina et al., 2002), their intensive study has the potential of discovering important environmental associations, as demonstrated by the experience with Western Pacific ALS. Noteworthy is that a cluster across generations might arise from a genetic factor, an established local practice, or a geographically restricted exposure (Malaspina et al., 2002).

#### France and Italy

Environmental factors were examined closely in a hotspot of sALS in Savoie in southeast France (Lagrange et al., 2021b). After excluding a role for heavy metals, pesticides, garden chemicals, and L-BMAA in drinking water, a case-control study of 14 sALS patients among part-time and full-time residents of a ski-resort hamlet revealed the prior food use of wild mushrooms (*Gyromitra* spp., including the Snow Morel *G. gigas*) (Miller et al., 2020). Subsequent investigation revealed that ALS patients also consumed the poisonous False Morel, *G. esculenta.* Half of the ALS cases reported an acute illness following ingestion of *gyromitres* 5–20 years prior to onset of muscle weakness. Control subjects had also collected and eaten wild mushroom species, but not *G. esculenta*. Banned for sale in France, *G. esculen*ta (False Morel, Brain mushroom, Turban fungus) contains gyromitrin (*N*-methyl-*N*-formylhydrazine) and eight additional homologous hydrazones that generate genotoxic MMH (Nagel et al., 1977; Liener, 1986; Trestrail, 1994, 2000) which, like cycad-derived MAM (Matsumoto and Higa, 1966; Nagata and Matsumoto, 1969; Nair, 1990) and nitrosamines (Shank and Magee, 1967) produces carbon (methyl) free radicals that methylate DNA and RNA.

**FIGURE 1:**
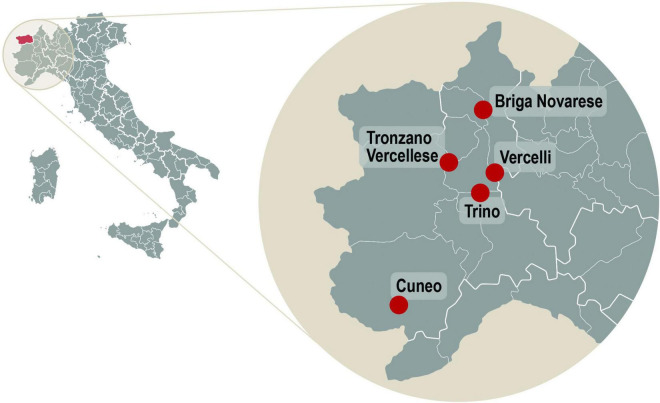
Left image: Map of Italy (including the islands of Sardinia **center left** and Sicily, **bottom**), showing the Aosta Valley in red **(upper left)** and nearby ALS hotspots **(right)** in Piedmont. The town of Acqui Terme is located ∼50 miles due east of Cuneo.

While the generality of the following observation is unknown, samples of *G. esculenta* collected in southern France at middle altitudes (900–1,200 m, Mt. Aigoual, Gard/Lozère; Espinouse Mountains, Hérault) reportedly have higher MMH concentrations (200–350 mg MMH) than concentrations (50–60 mg MMH) found in specimens at higher altitudes (2,200 m) in the Pyrénées Orientales, France (Lac des Bouillouses). Prolonged desiccation (76 days for > 6 months) resulted in the concentration of MMH stabilizing around 300–450 mg/kg of dry specimen (equivalent to about 15–30 mg MMH/kg of reconstituted fresh specimen) (Andary et al., 1985). Collection of wild mushrooms for food is locally popular on the other side of the Alps, in northwest Italy (Piedmont, Aosta Valley) (Anon, 2022t); ALS incidence is high (Marin et al., 2017), and hotspots of sALS have been reported in Cuneo and Vercelli (Migliaretti et al., 2013), Briga Novarese, Trino and Tronzano, and Vercellese (Uccelli et al., 2007; Figure 1), and confirmed in Acqui Terme, province of Alessandria (*vide infra*) (Vasta et al., 2018), regions (province of Biella) that harbor MMH-generating fungi, notably *G. esculenta* (Pers.) Fr. (Anon, 2022n), that can trigger acute poisoning (Andary et al., 1985; GBIF, 2022). Today, food use of *Gyromitra* spp. in Piedmont is reportedly almost non-existent (Nicola Sitta, personal communication, February 2022).

**FIGURE 2:**
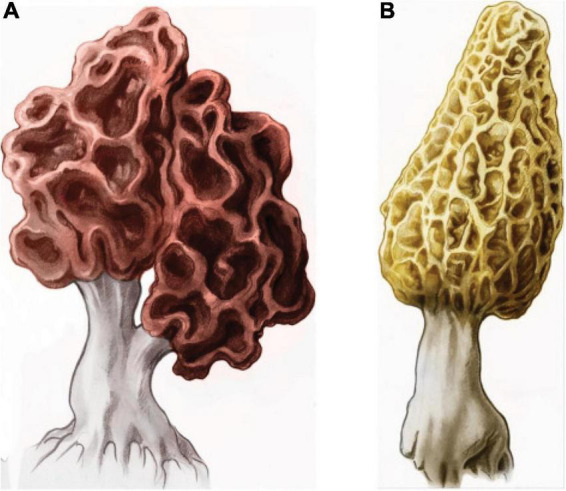
**(A)** False Morel (*Gyromitra esculenta*). **(B)** True Morel (*Morchella esculenta*). Reproduced from Arłukowicz-Grabowska et al. (2019) under Creative Commons license. Mistaken identification of morels can lead to group poisoning events, as occurred in February 2019 among diners at a Michelin-starred restaurant in Valencia, Spain. https://www.thelocal.es/20190221/michelin-starred-valencia-restaurant-closed-amid-fears-diner-died-from-poisonous-mushroom/. In 2020, the U.S. issued an alert given the history of *G. esculenta* contaminating imported commercial shipments of dried and canned *M. esculenta* (FDA, 2020). A 2-year survey showed 21% of the True Morel and 15% of the wild mixed mushrooms were contaminated with toxic look-alike species (Gecan and Cichowicz, 1993). An epidemiologic study of ALS in southern Greece showed an overrepresentation of farmers among patients and an aggregation of cases in the region of Cephalonia (Kalfakis et al., 1991), where morels occur (Denchev et al., 2013). See also: how to identify a real morel from a false morel. Available at: https://www.youtube.com/watch?v=OXePLfjCUeI.

On the island of Sardinia (Figure 1), the distribution of ALS was non-homogenous (more cases in rural areas) and more common among farmers and shepherds with low levels of education (Giagheddu et al., 1983, 1993; Grainieri et al., 1989). While no association has been made between ALS and food use of mushrooms outside of southeast France, wild morels are among the fungi collected in Sardinia (Anon, 2020), and many authors have warned that some MMH-generating False Morels can be mistaken for the highly sought-after True Morel (*Morchella esculenta*) (Gone 71°N, 2022; Figure 2). In recent decades, there has been a sharp rise in foraging for wild mushrooms across Sardinia along with an increase in the number of acute (but unspecified) intoxications, few of which have proved fatal (Comandini et al., 2018). Noteworthy is that images #11 and #21 in an historical treatise on the hypogeous fungi of Sicily and Sardinia (Mattirolo, 1900) depict False Morels (*Gyromitra* spp.).

#### Denmark, Sweden, and Finland

The incidence and prevalence of ALS is high in the Danish Faroe Islands (Johansen et al., 2022), particularly the southernmost island of Suðuroy, where the prevalence is three times higher than the nationwide prevalence (Johansen et al., 2021). While familial clustering (14%) was in excess of that expected for ALS on Suðuroy, a result suggestive of genetic contribution, environmental factors were not excluded or sought. In Sweden, compared to the rest of the population, agricultural work was significantly more common among cases in an ALS cluster in the county of Skaraborg (Gunnarsson et al., 1996), where *G. esculenta* was formerly eaten (Denchev et al., 2013; Svanberg and Lindh, 2019). Additionally, as discussed elsewhere (Spencer, 2019), the birth location of a cluster of ALS subjects in Finland (Sabel et al., 2003) corresponded to a region of False Morel consumption promoted by wartime-associated food shortages. Between 2000 and 2015, almost 20% of mushroom-identified calls to the Finnish Poison Information Center involved acute food poisoning from *G. esculenta* (Tähkäpää et al., 2020).

#### United Kingdom

Clusters of ALS have been reported in areas of southeast England and East Lancashire (Mitchell et al., 1990, 1998; Scott et al., 2009). *G. esculenta*, which has a localized U.K. distribution in coniferous regions, has been reported in these regions (Anon, 2022j,o). The East Lancashire focus of ALS in Addington/Owaldtwistle is the location of the urban Foxhill Nature Reserve where mushroom hunters are taught to identify poisonous species (Cruces, 2010). The U.K. lists 4 *Gyromitra* spp. and 36 *Agaricus* spp. (BMS, 2022), the latter containing various concentrations of the phenylhydrazine derivative agaritine [*N*-(γ-L(+)-glutamyl)-4-hydroxymethylphenylhydrazine] (Schulzová et al., 2009).

#### Spain

A 2018 study identified three ALS clusters in agriculture-intensive areas of north/northeast Catalonia (Povedano et al., 2018), two of which are proximate to national parks with edible True Morels (*M. dunalii*) and several poisonous hydrazinic fungi (*Agaricus* spp.) (Sierra and Valverde-Valera, 2019), one of which [*A. bitorquis* (Qu l.) Sacc.] had (in the Czech Republic) a very high content of agaritine (Schulzová et al., 2009). The Catalonian authors proposed their results were consistent with exposure to agricultural pesticides, as well as to air pollution, but dietary factors were not addressed. Among wild mushrooms in Catalonia are the *bolet de greix* (fat mushrooms), namely *G. esculenta and G. gigas*, that grow beneath pine trees during Spring, and *G. infula*, which is found in the autumn (Anon, 2011). *G. esculenta*, while recognized to be poisonous, reportedly has been consumed since ancient times in the Valleys of the Pyrenees (Anon, 2022e). In recent times, *G. esculenta* has been sold in a market called *Mercat del bolet de Cal Rosal* located in Olvan (Bergueda) (R.V.A., personal observation), which joins the Pyrenees and Central Depression and is within the north-south cluster of ALS in the Barcelona region. Further north in the Pyrenees, *G. esculenta* and *G. infula* are recorded in the Ordesa y Monte Perdido National Park (Pancorbo et al., 2017).

#### Southwest Central France

Three ALS clusters in Nouvelle Aquitaine (formerly Limousin) were geostatistically linked to the presence of paper paste and water-treatment plants; the authors suggested that heavy use of chemicals and water in these plants would create habitats favorable to cyanobacteria and, hence, the generation of L-BMAA (Boumédiène et al., 2011). Formaldehyde is heavily associated with paper or paperboard production, while hydrazine is mainly used for eliminating oxygen in water for steam generation in the paper industry (Korhonen et al., 2004). Hydrazine is widely employed in thermal engineering as an anticorrosive agent; when preserving and passivating equipment, a large volume of wastewater containing a high concentration of hydrazine (about 100 mg/dm3) is formed after chemical cleanings (Gogolashvili et al., 2001). In the Limousin study, the Standardized Incidence Ratio for ALS exceeded unity (but without statistical significance) for water-treatment stations that used an active sludge system, which cannot handle hydrazine (Farmwald and MacNaughton, 1981). Also noteworthy is the strong mushroom culture in Limousin that includes distinguishing edible (notably *Boletus edulus*, or *cèpe*) from toxic varieties (Harley, 2021), including *G. esculenta* (Taylor, 2008). A 2003–2011 population-based study of >5 million inhabitants in 10 *départements* (the name assigned to the largest unit of government in France) in 5 regions of France found the “possible over-incidence” of ALS (8 cases vs. 2 expected) in one *département* (Haute Vienne, town of Rochechouart) of Nouvelle Aquitaine (Boumédiene et al., 2022). Among seven areas subject to complete the robust analysis, only one “definite cluster of ALS” was identified, namely that associated with *gyromitres in* the French Alps (Lagrange et al., 2021b).

#### United States Mainland

**FIGURE 3:**
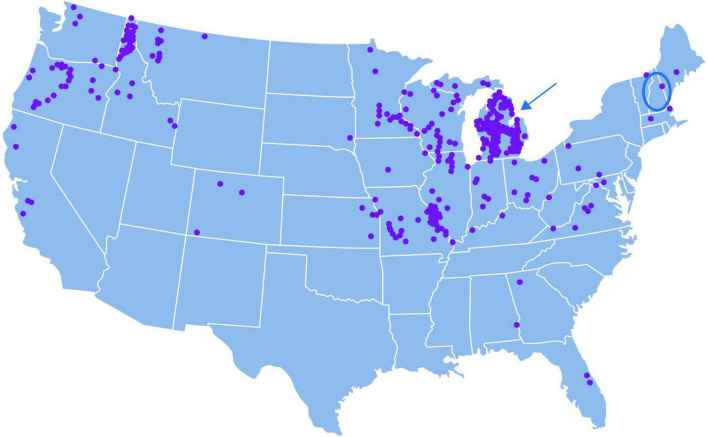
Map of USA in which each dot represents one call to a U.S. poison center regarding ingestion of a mushroom species containing monomethylhydrazine (MMH) over the 10-year period 2001–2011. Redrawn from Hatten et al. (2012). A 2006 paper from the North American Mycological Association tabulated reports of acute illness from *Gyromitra* spp. (including *G. brunnea, esculenta, gigas, montana*) from Arkansas, Idaho, Iowa, Massachusetts, Michigan, Montana, Quebec, Oregon, and Washington (Beug et al., 2006), and an Asian couple west of the North American Rockies (Leathem and Dorran, 2007). Several MMH reports originated from the northwestern United States (notably Oregon, Idaho), northcentral and central states, including Wisconsin (adjacent to Michigan) Missouri (southernmost cluster of dots) and a few from New England states, including New Hampshire (*oval*). **(Upper-right)** The highest concentration of MMH-related calls came from Michigan (*arrow*) in the Great Lakes region of the upper Midwestern United States, the home of several species of poisonous False Morels (Mushroom Observer, 2015). Areas of Michigan suspected to have a high ALS incidence include the towns of Cadillac and Greenville, and Newaygo County (Anon, 2022b). Mesick, a town 19 miles northwest of Cadillac, holds morel-hunting contests at the May Annual Mesick Mushroom Festival (Anon, 2022p). The National Morel Mushroom Festival takes place in Boyne City in northern Michigan (National Morel Mushroom Festival, 2022). These locations are all in northern Michigan’s Lower Peninsula where wild morels have been mapped (Morels, 2022). Note that fungi take up not only metals from soil but also persistent environmental pollutants, including organochlorine pesticides, polychlorinated biphenyls, and brominated flame retardants (Moeder et al., 2005; Rodríguez-Rodríguez et al., 2012; Bokade et al., 2021), certain members of which were elevated in the blood of Michigan patients with ALS (Su et al., 2016).

MMH-related mushroom poisoning is recorded in many countries, including the USA, notably the State of Michigan (Trestrail, 1994; Hatten et al., 2012; Brandenburg and Ward, 2018; Horowitz et al., 2022; Figure 3) in the Midwest, which has among the highest prevalence of ALS in the nation^2^. Self-identified clusters of ALS are common in Michigan, such as three friends with proximate childhood homes who later developed ALS around the same time (Feldman, 2000). A case-control study of ALS in Michigan (Yu et al., 2014) that sought information on occupational and residential exposures, residence location, exercise and sports, body weight, tobacco use, military experience, and family history, found an association with fertilizers and pesticides and no association with smoking, occupational exposures to metals, dust/fibers/fumes/gas and radiation, and physical activity. Questions related to diet were not included in this study.

A 1989 study found evidence for clustering of ALS in northeast Wisconsin adjacent to northwestern Lake Michigan (Taylor and Davis, 1989; Figure 3), where wild morel season begins in May (Anon, 2022d); while *G. esculenta* can be found (Anon, 2022l), *G. brunnea* is most common (Volk, 2002; Anon, 2022l). Additionally, a 1990 case-control study found a small cluster of ALS cases among long-term residents of Two Rivers, Manitowoc County, Wisconsin. Physical trauma, the frequent consumption of freshly caught Lake Michigan fish, and a family history of cancer were reported more often by case patients than control subjects (Sienko et al., 1990). While diet was among many factors examined, it is not clear whether their research instrument queried the popular practice in Wisconsin of collecting and eating wild morels (Craddock, 2017; Anon, 2022f).

A 2009 study of nine patients in Enfield, New Hampshire (Figure 3), revealed an incidence of sporadic sALS that was 10–25 times the expected incidence of 2/100,000/year (Caller et al., 2009, 2013). The patients lived close to Lake Mascoma, one of several New Hampshire waterbodies with higher-than-average mercury concentrations (Neils and Nelson, 2018) and a history of algal blooms. The authors suggested the ALS hotspot might arise from chronic exposure to cyanobacterial neurotoxins (such as L-BMAA) in association with aerosols, fish consumption or ingestion of lake water. Uninvestigated is whether these cases engaged in the Spring harvesting of True Morels (*M. esculenta*) (Anon, 2016; Figure 2) but collected and consumed *G. esculenta* either deliberately or in error (De Román et al., 2006), a subject addressed by the Northern New England Poison Center (Colin, 2019). Other ALS clusters have been reported in eastern and northwestern Vermont, Maine and western New Hampshire (Caller et al., 2013), where seasonal collection of wild morels is also popular (Garrett, 2012; Anon, 2022r).

A 2008 epidemiologic investigation of ALS in Jefferson County, Missouri (Figure 3), identified a small cluster of patients around a lead smelter area (Taylor and Davis, 1989). Mushrooms flourish in smelter areas, where they take up heavy metals such as lead (Spencer and Palmer, 2021). In the case of *G. caroliniana* (Big Red) (Kuo, 2021), which is distributed statewide in Missouri (Anon, 2022c), fungal metal uptake might require continuous production of hydrazine-related compounds to store the potentially toxic elements in the form hydrazine-metal chelates (Govindarajan et al., 1995). Selenium is of interest in this regard because this metal has been linked to ALS in South Dakota USA (where *Gyromitra* spp. also occur Miller et al., 2020) and in Reggio Emilia Italy (Kilness and Hichberg, 1977; Vinceti et al., 2010a,b), where education on False Morels has been posted (Cocchi and Siniscalco, 2022). *G. caroliniana* is widely available in North Carolina, where bulbar presentation of ALS occurred in three geographically proximate long-term residents (Hochberg et al., 1974).

### Conjugal ALS

Conjugal cases are important because of the possibility of identifying a history of common environmental exposures with potential relevance to etiology (Godeiro-Junior et al., 2009), as demonstrated by the association of cycad toxins with Western Pacific ALS, especially on Guam (Spencer et al., 2020). Outside of Guam, where conjugal cases of ALS were reported in 1975 (Reed et al., 1975), diagnoses of ALS in couples is rare (Dewitt et al., 2012). Through 2021, there were reports of at least 20 conjugal ALS pairs in the literature. There was an important geographical cluster of 10 pairs in southeast France (Camu et al., 1994; Corcia et al., 2003; Lagrange et al., 2021b), 5 pairs in Italy (Paolino et al., 1984; Poloni et al., 1997; Rachele et al., 1998; Chiò et al., 2001; Bersano et al., 2014; Vasta et al., 2018), 2 pairs each from Brazil (Godeiro-Junior et al., 2009) and the UK (Orrell et al., 1996; Fernandes et al., 2017), one pair each from Libya (Maloo et al., 1989) and Spain (Martínez Matos et al., 1986), and 4 pairs from the United States (Chad et al., 1982; Cornblath et al., 1993; Palmer and Spencer, 2002). No conjugal case was shown to be consanguineous and, prior to ALS diagnosis, all pairs had lived together for at least 10 years and sometimes much longer. These reports raise the possibility of shared exposure to unknown environmental risk factors; unfortunately, they have been often dismissed as coincidental, random associations.

#### France

**FIGURE 4:**
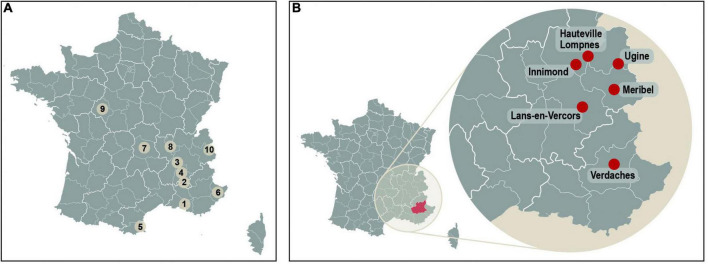
**(A)** Geographic distribution of the conjugal amyotrophic lateral sclerosis cases. The French territory is divided on the map into its different *départements*. Each conjugal case is represented by its number in the text. #1, Bouches-du-Rhône; #2-4, Drôme; #5, Pyrénées-Orientales; #6, Alpes-Maritimes, #7, Puy-de-Dôme; #8, Rhône; #9 Indre-et-Loire, #10 Montchavin-Les Coches. Modified from Corcia et al. (2003) to include data from Lagrange et al. (2021b). **(B)** Location of genetically confirmed samples of *G. gigas* Krombe. Cooke collected from woodland conifers in S.E. France. Left: Alpes-de-Haut-Provence, Colmars, Ratery. Right: Ain, Hauteville-Lompnes, Col de la Berche; Ain, Innimond, Plaine du Bief; Isere, Lans-en-Vercors, Combe de Servagnet; Savoie, Ugine, La Mollette; Savoie, Les Allues, Altiport de Méribel; Alpes-de-Haut, Verdaches, Haut-Bès. (Sourced from the Illinois Natural History Survey Fungarium, University of Illinois, January 2022).

Two reports of geographic clustering of conjugal ALS cases in southeast France are of singular importance (Camu et al., 1994; Corcia et al., 2003; Figure 4A). In total, 18 patients representing 9 couples (cases) presented with ALS between January 1975 and December 1999. Eight patients had disease onset between 1975 and 1992, while 10 were diagnosed during or after 1994. The mean age of onset was 65 years (range, 41–85 years), and the mean interval between onset of spousal ALS was 8 years (range, 1–19 years). Disease onset was spinal in 60% and bulbar in one third. There was no known consanguinity between affected spouses, and there was no major predominance of a given occupation or any specific environmental exposure that could be identified. The mean conjugal lifetime before the first ALS case was 10 to > 40 years (mean: 25 years), which is consistent with the long-latent period for post-exposure development of Western Pacific ALS (Spencer et al., 2020). Three of the conjugal cases resided in Drôme *département* and two of these lived in Valence, a town in Auvergne-Rhone-Alpes not far removed from the *département* of Savoie to the northeast, the location of a cluster of ALS patients in Montchavin (Lagrange et al., 2021b), including a conjugal case (Figure 4A), all of which reported a history of food use of *gyromitres*. The University of Illinois Natural History Survey Fungarium lists genetically confirmed examples of *G. gigas* in the S.E. French alpine region (Figure 4B); an equivalent map for *G. esculenta* is not yet available.

#### Italy

One of the 5 reported conjugal Italian cases resided in a hotspot of ALS in Acqui Terme, a town situated in the Monferrato area of the province of Alessandria, Piedmont, Italy (Bersano et al., 2014; Vasta et al., 2018; Figure 1, right). In 2005, spinal-onset ALS was diagnosed in a 63-year-old male and, 3 years later, bulbar-onset ALS in his 68-year-old non-consanguineous wife. Genetic screening of both patients revealed no ALS-associated mutant genes. Environmental histories identified no common exposures to radiation, food-borne pathogens, cosmetics, drugs, or pesticides in agricultural environments; nor were there exposure risks from smoking, intense physical activity, or trauma. While the etiology was not identified, it is noteworthy that a May 16, 2020 article in the local Acqui Terme newspaper *L’Ancora* described an undefined syndrome associated with food use of *Gyromitra* spp. (*Falsa Spugnola*), which contains high concentrations of “*giromitrina* (a hydrazine mixture).” An article on page 9 described two “*Serate Micologiche*” (Evenings Mycological) devoted to fungal toxicology organized from the Punto Cultura Association, with the patronage of the municipality of Acqui Terme and the Province of Alexandria.

Conjugal ALS was also reported to affect a married couple, members of which originated from different regions of Italy and lived in a small Piedmont town of 19,571 inhabitants (Chiò et al., 2001), a population approximating that of Acqui Terme (Figure 1, right). The husband was diagnosed with ALS in 1994 (age 61 years), the wife in 1999 (age 53 years). He had used “solvents: nitro-compounds and dimethylketone.” Prior to their diagnoses, both had engaged in various jobs including, between 1989 and 1993, the collaborative maintenance and operation of a gasoil-powered central heating system that served the apartments of the house in which they resided. Their chemical exposure during this period was unstated, but it is of potential interest that hydrazine solutions are used to control oxygen corrosion in boiler systems (Scrivenand and Winter, 1978).

Three other Italian reports describe non-consanguineous conjugal cases of ALS in the second half of life. Two conjugal pairs involved residents of Sardinia (Figure 1, left). One pair (from Barbagia, province of Nuoro) was involved in sheep breeding (Paolino et al., 1984), another lived next to a distillery (Rachele et al., 1998). The third pair lived adjacent to a metallurgical plant in a heavily farmed area of southern Italy (location not stated). Affected couples had lived together for 3–4 decades (Poloni et al., 1997; Rachele et al., 1998), with common exposure to water and garden vegetables reportedly resulting in no significant exposure to heavy metals, pesticides or “known toxic substances” (Poloni et al., 1997). Couples were considered to have conjugal ALS by chance (Paolino et al., 1984; Rachele et al., 1998; Chiò et al., 2001).

#### Other countries

Three papers describe three conjugal cases of ALS in mainland USA (Chad et al., 1982; Cornblath et al., 1993; Palmer and Spencer, 2002), but none investigated common environmental exposures and one attributed the conjugal association to chance (Cornblath et al., 1993). A conjugal case occurred in a >20-year married couple; the husband worked for the U.S. military as an electrical mechanic with large air conditioners and refrigeration and had chemical-saturated clothing washed by his wife (Palmer and Spencer, 2002). Common exposures were also not explored for (a) an elderly Libyan couple who had lived together for 40 years (30 years in Benghazi) and developed ALS within 15 months of each other (Maloo et al., 1989) or (b) for another conjugal ALS couple from Spain (Martínez Matos et al., 1986). Another study lacking an exposure history described two couples with conjugal ALS from Brazil; one couple had lived together for 40 years, and the second for 20 years in the countryside outside Sao Paulo (Godeiro-Junior et al., 2009). Near simultaneous onset of motor weakness occurred in the 7th decade of life of a couple living in Scotland; since no common environmental history was found, conjugal ALS was assumed to have occurred by chance (Fernandes et al., 2017). Studies of ALS in the Lothian lowlands of Scotland between 1961 and 1981 did address potential environmental exposures to the extent of employment history (Holloway and Mitchell, 1986). None of the foregoing studies explored food-related exposures.

### Twins discordant for ALS

Twins with only one ALS-affected subject can serve as matched-pair case-controls for study of disease etiology (Schlesselman, 1982). A 1997 British (England and Wales) study of 70 pairs of monozygotic and dizygotic twins assessed the environmental exposure history of pairs of twins discordant for ALS (Graham et al., 1997). Seventy-seven probands were identified, of which 26 were monozygotic and 51 dizygotic, with deaths between 1979 and 1989. Four monozygotic probands were concordant, but two probands came from a family known to have familial ALS. The content of the questionnaire and interviews of surviving relatives was not stated, but the results suggest the authors focused on occupation. Although analysis of the exposure history of discordant monozygotic twins would be of primary interest, small subject numbers required the authors to include both discordant monozygotic and dizygotic twins in their analysis. The strongest and most highly significant association (OR 7.0, 95% CI 1-33-89-90, *p* = 0.006) was with a history of regular car/vehicle maintenance (Graham et al., 1997). Noteworthy is that hydrazine was used to fuel racing cars, funny cars and dragsters in the 1960s (with events in the U.S. and U.K.) (Cook, 2018; Anon, 2022a), and several individuals with a history of motor racing developed ALS (Spencer, 2019), as did a Gulf War Veteran with familial ALS (Palmer and Spencer, 2002). The British twin study also found a significant association between motor neuron disease and a history of occupational paint usage (OR = 3.75; 95% CI 1-0-17 1, *P* = 0.022) (Graham et al., 1997). Hydrazide compounds are widely used in paint and adhesive thermoset applications, including latent hardeners for epoxy resins and as crosslinking agents in acrylic emulsions (Hara, 1990; ATSDR, 1997).

### Close-proximity ALS groups

There are several brief reports of unrelated people living or working in close proximity who developed ALS at approximately the same time (Table 1).

**TABLE 1:** Close-proximity ALS case groups.

	ALS grouping	Location	Common exposures	Environmental factors	References
A	Ranchers (*n* = 3)	South Dakota, USA	Proximate residences	High selenium soil content	Kilness and Hichberg, 1977
B	Males (*n* = 3)	North Carolina USA	Common residence	No association proposed	Hochberg et al., 1974
C	Mail clerks (*n* = 3)	Florida, USA	A11 died within 10-years period	No association proposed	Sanders, 1980
D	Three (2M, 1F)	Montreal, Canada	Apartment complex	No association proposed	Melmed and Krieger, 1982
E	Teachers (*n* = 3)	Ohio, USA	Same classroom	No association proposed	Hyser et al., 1987
F	NASA staff (*n* = 7)	California, USA	Common workplace	No association proposed	Noack, 2016; NASA Ames Astrogram, 2018
G	Population	Missouri, USA	Lead smelter proximity	Small cluster	Turabelidze et al., 2008
H	Three friends (3F)	Michigan, USA	Childhood proximity	No association proposed	Feldman, 2000

Rows: (A) Region has a high soil content of selenium, one of many elements taken up and concentrated by mushrooms (Spencer and Palmer, 2021). (B) Unstated occupational, travel, dietary and animal-exposure histories similar to that of local people. Hunting for True Morels and avoiding False Morels is an annual pastime between March and May in North Carolina (Johnson, 2020). (C) G. *montana* was sold to unnamed restaurants in Florida (Bergo, 2021) and poisonings from unnamed wild mushrooms are recorded in the State (Kintziger et al., 2011). (D) Ashkenazi Jews. Wild mushrooms, including morels, are considered kosher and, as such, have served as an important component of food for Ashkenazi and other Jewish people (Gelman, 2019). (E) No unusual shared dietary habits or medications, different residential locations, and no other social, occupational or environmental interactions. Hydrazine-related chemicals may be used in schools (EPA, 2006; NIOSH, 2006). (F) The National Aeronautics and Space Administration (NASA) Ames Research Center led development of the liquid hydrazine-propelled *Pioneer 10* spacecraft launched in 1972 (NASA, 2022), and hydrazine sulfate and ammonium hydrazinium sulfate were subjects of five papers listed by Ames published between 1993 and 1998 (Spencer, 2019). (G) A small but significant cluster (*p* = 0.04) was detected around the lead smelter area (Wang et al., 2014; Meng et al., 2020); lead is taken up by mushrooms (Spencer and Palmer, 2021). (H) Three friends whose childhood homes were located proximate to the County High Point of Kalamazoo, Michigan. Kalamazoo is a popular location for collection of wild morel mushrooms (see: https://www.mlive.com/news/kalamazoo/2016/04/planning_a_morel_mushroom_hunt.html).

A 2007 report described three friends who grew up in the same village in southeast England, began to play soccer at age 15/16, continued together at a moderately high level for many years (sometimes in the soccer league/team), and 10–20 years after they had stopped playing soccer, developed symptoms of ALS (no family history of ALS) within a few years of each other (Wicks et al., 2007). This account was preceded and followed by reports of an elevated risk of ALS among Italian First and Second Division soccer players. Whereas no cases of ALS were found during the 1970–1979 period, the standardized morbidity ratio was significantly increased for the periods 1980–1989 and 1990–2001. Moreover, a dose-response relationship between the duration of professional football activity and the risk of ALS was found (Chiò et al., 2005), with a younger age at onset of symptoms in soccer players born in more recent years (Vanacore et al., 2018). The mean age at diagnosis was 45.0 years, > 20 years earlier than that for the general population (Pupillo et al., 2020). No cases of ALS were found among professional basketball players or cyclists, suggesting that the physical activity of soccer players *per se* was not causally related (Chiò et al., 2009), although a possible role for repetitive head trauma during play could not be excluded (Beghi, 2013). The Italian and English reports also raised the possibility of repetitive player contact with pesticidal chemicals used on soccer pitches (Vanacore et al., 2006).

## Discussion

We propose intensive study of young-onset, conjugal, twin-discordant and clusters of sALS as a method to discover and identify exogenous agents with the potential to trigger motor neuron disease. Among such agents, acting alone or in the presence of a genetic susceptibility, there is evidence that sufficient exposure to naturally occurring or synthetic hydrazine-related chemicals is associated with the development of clinical ALS years or decades later. While this association requires confirmation, it is known that hydrazines and MAM form carbon-centered free radicals (potent alkylating agents) that can methylate DNA in the *O*^6^-, N7-, and C8-positions of guanine (Kobayashi and Matsumoto, 1965; Matsumoto and Higa, 1966; Shank and Magee, 1967; Albano et al., 1989; Gamberini and Leite, 1993; Kisby et al., 1999; Spencer et al., 2015). The accumulation of DNA lesions is responsible for the teratogenic, mutagenic, hepatotoxic, carcinogenic, and neurotoxic properties of MAM (Laqueur, 1977; Sieber et al., 1980; Kisby and Spencer, 2011). Neurons are proposed to be susceptible to *O*^6^-methylguanine because the specific DNA-repair enzyme *O*^6^-methylguanine methyltransferase (MGMT) is downregulated in mid to late S-phase of the cell cycle (Mostofa et al., 2018) such that post-mitotic cells have low MGMT levels (Kisby and Spencer, 2021). Noteworthy is that changes in gene and protein expression of MGMT have been found in Alzheimer disease (Oláh et al., 2015; Chung et al., 2022; Kisby et al., 2022).

### ALS and botanical exposures to hydrazinic chemicals

**FIGURE 5:**
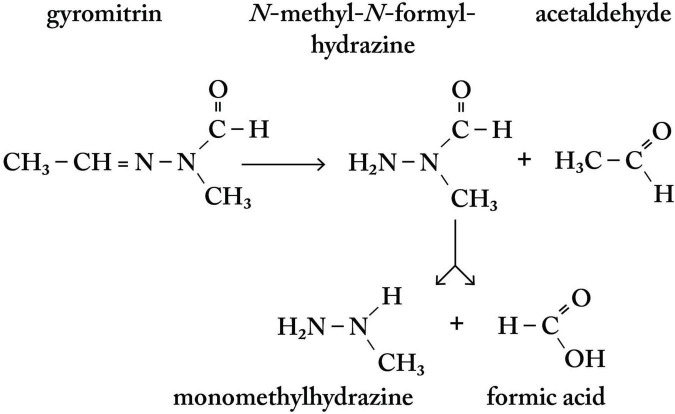
Metabolic pathway to monomethylhydrazine (MMH) of the major hydrazone gyromitrin in *G. esculenta*. MMH has acute neurotoxic properties but, like cycad-derived methylazoxymethanol, also induces DNA lesions (*O*^6^- and N^7^-methylguanine) that are poorly repaired in neurons and are proposed to result in multiple downstream cellular effects linked to neurodegenerative diseases, such as ALS.

Fungal hydrazine-related compounds include agaritine in *Agaricus* spp. (Schulzová et al., 2009) and gyromitrin in *Gyromitra* spp. (Monographs, 1987; Figure 5) and certain other fungal genera (*vide supra*) (Trestrail, 2000). The association between food use of *gyromitres* and sALS in Savoie, France (Lagrange and Vernoux, 2020; Lagrange et al., 2021b) receives support both from the discovery of multiple conjugal cases clustered in adjacent French *départements* and in pockets of ALS to the east in Piedmont, Italy, where *gyromitres* are consumed. Since *G. gigas* reportedly contains 1,500-fold lower concentrations of gyromitrin than that of *G. esculenta* (Viernstein et al., 1980), the latter constitutes a substantially greater health threat and, thus, this highly poisonous species may have contributed to ALS cases in both Savoie and Piedmont, Italy. Urgently needed are precise analytical methods (e.g., HPLC-MS/MS) to quantify gyromitrin and minor hydrazones in *Gyromitra* spp. under specified environmental conditions. Focused research is merited in these regions to test this hypothesis in retrospective and, potentially, prospective studies. There is also justification to explore whether other pockets of sALS are linked to deliberate or mistaken food use of False Morels (Cruces, 2010; Beug, 2014; Pulse, 2022), especially in northern Michigan, USA, given the State’s relatively frequent occurrence of acute MMH poisoning (Figure 3) and high prevalence of ALS (Feldman, 2000). In Europe and Scandinavia, most acute MMH intoxications occur from consumption of False Morels collected in the conifer forests of Germany, Poland, Sweden, and Finland (Horowitz et al., 2022). The birth location of a cluster of ALS subjects in Finland (Sabel et al., 2003) corresponds to a region of False Morel consumption (Spencer, 2019). *Gyromitra esculenta*, among other species, if found on conifers (mostly pines) across Europe, Scandinavia, and North America, parts of Central (Mexico, Costa Rica) and South America (Argentina, Chile) and the Caribbean (Dominican Republic, U.S. Virgin Islands), parts of Asia (Georgia, Japan, Kazakhstan, Pakistan, Russia), including India (Jammu and Kashmir) and China (Heilongjiang), Northern Africa (Algeria, Morocco, Canary Islands), Australia, and New Zealand (Minter, 2018).

There are numerous factors that determine the toxic effects of fungal hydrazones in False Morels (Nordic Council of Ministers, 1995). The concentration of the principal hydrazone gyromitrin (Figure 5) is said to vary across *Gyromitra* species, age, geographic location, elevation, temperature (Anon, 2022q) and, conceivably, the soil content of metal elements and atmospheric humidity. All parts of the fungus are potentially toxic, including the stipe and cap that are used as food. The concentration of gyromitrin changes according to the method and duration of mushroom preservation (refrigeration, canning, desiccation) (Pyysalo, 1976). Fresh European specimens of *G. esculenta* may contain 1,200–1,600 mg/kg gyromitrin, while concentrations in dried tissue have ranged between 14.7 to >6,400 mg/g (Nagel et al., 1977). False Morel food preparation (cutting, washing, boiling, frying) reduces gyromitrin concentration and, since MMH boils at 87.5°C, fumes with potential for acute human intoxication are released into the air (Anon, 2022j). The amount, frequency and duration of consumption determines the dosage of hydrazones received by the consumer and the total amount of MMH (via *N*-methyl-*N*-formylhydrazine) generated by acid hydrolysis in the stomach (Nagel et al., 1977; Garnier et al., 1978). Symptoms of acute MMH intoxication appear 6–14 h after ingestion or 2–8 h after inhalation, with the lethal dose in micrograms/kg estimated to be 10–30 for children and 20–50 for adults (Trestrail, 2000). Whatever the route of exposure, gastrointestinal illness may be accompanied by hemolysis and, in severe intoxication, hepatorenal toxicity with jaundice, liver failure, delirium, seizures, coma, and death (Trestrail, 2000).

Individual susceptibility to acute MMH toxicity seems to vary widely (Miller et al., 2020), but no formal studies have been carried out to establish this widely held impression. The toxin affects the liver, central nervous system, and sometimes the kidneys. As with MAM-liberating cycad seed, acute food poisoning from ingestion of *G. esculenta* takes the form of vomiting and diarrhea several hours after consumption, often followed by dizziness, lethargy, vertigo, tremor, ataxia, nystagmus, and headaches and fever. Severe cases may exhibit delirium, muscle fasciculation and seizures, mydriasis progressing to coma, circulatory collapse, and respiratory arrest 5–7 days of ingestion (Michelot and Toth, 1991). Acute poisoning by *Gyromitra* spp., which is similar in character to the toxic effects of hydrazine propellants (Azar et al., 1970), is scarce in Western Europe and more frequent in Eastern Europe (Patocka et al., 2012; National Research Council [NRC], 2022).

In other alpine countries (Switzerland, Monaco, Italy, Liechtenstein, Austria, Germany, and Slovenia) and beyond (Eastern Europe, Russia), *G. esculenta* has various local names (Michelot and Toth, 1991; Anon, 2022i; GBIF, 2022) and mushroom culinary traditions vary (Peintner et al., 2013). Other fungi reported to contain gyromitrin include (*Cudonia circinans* syn. *Leotia circinans*), *L. lubrica* (Jelly Baby), *Helvella crispa* (Elfin Saddle), *H. lacunose* (Slate Gray Saddle), *H. elastica* (Elastic Saddle), and *H. macropus* (Felt Saddle) (Andary et al., 1985; Flume, 2022).

Wild hydrazinic mushrooms consumed in Sicily include *H. crispa* (*spugnola crespa, funci di chiddi rizzi, munachessi*) and two species of *Agaricus* (*funcia campagnola, funcia picurina*) are eaten raw or cooked (Lentini and Venza, 2007). This may be relevant to the spatio-temporal and spatial high-incidence clusters of sALS on the southeastern flank of Mt. Etna in Sicily that have been linked to a possible etiologic role for metals in volcanic ash (Nicoletti et al., 2016; Boumediene et al., 2019). Importantly, fungi are able to accumulate metals from contaminated soils and even have a role in soil bioremediation (Spencer and Palmer, 2021).

There appears to be a narrow margin for ingestion of *Gyromitra* spp. between individual tolerance and development of acute illness; symptoms may follow an “all or none” pattern both in monkeys and humans (Miller et al., 2020; National Research Council [NRC], 2022). Nothing is known about individual susceptibility, but age, physical health and gene status are possible variables, such that individuals with fast acetylator hepatic metabolism may be able to mitigate the potential acute and delayed effects of consuming False Morels (Lagrange and Vernoux, 2020). Genetic variation in expression of microsomal cytochrome P450 isoenzymes also has relevance since (in rat liver) hydrazine is metabolized and detoxified by CYP2E1, CYP2B1, CYP1A1/2, ultimately yielding molecular nitrogen (IARC, 1999). Subject medication may be another variable (Lagrange and Vernoux, 2020) since hydrazine-derived drugs also generate DNA lesions (Mathison et al., 1994) and alter microbiome composition (Wei et al., 2010). Vitamin B6 status is also significant since gyromitrin binds to and inhibits pyridoxal phosphokinase, the enzyme responsible for transforming dietary pyridoxine into active pyridoxal 5-phosphate, without which glutamic acid decarboxylase cannot convert glutamate to the neurotransmitter γ-aminobutyric acid (GABA). The resulting depletion of GABA promotes CNS excitation and seizures (Horowitz et al., 2022). While severe MMH poisoning from consumption of *Gyromitra* spp. is well documented, some people can consume appropriately prepared False Morels for years/decades without experiencing acute illness (List and Luft, 1967; Trestrail, 1994). Indeed, in Finland, local *G. esculenta* are commercially available for sale to the public according to strict regulations set by the Ministry of Trade and Industry of Finland and accompanied by precise food preparation instructions from the Finnish Food Authority (FFA) (Anon, 2022j; Pulse, 2022). Given that gyromitrin residues may remain when these instructions are followed, the FFA advised in 2019 against consumption by pregnant and breast-feeding women, and children (FFA, 2022). This appears to have been driven by concerns relating to the long-recognized carcinogenic and teratogenic potential of fungal hydrazines in laboratory animals (Toth, 1991, 1993; Toth and Gannett, 1994). Outside of Scandinavia, the sale of False Morels is generally banned (Germany, Switzerland) but still are consumed by some in Bulgaria and Spain,^3^ and they are available on-line for purchase from Piliakalni, Lithuania (Morel Mushrooms, 2022).

The foregoing makes it apparent that as-yet-undefined conditions must be fulfilled before the toxic properties of MMH are expressed clinically as an acute illness and, presumably, in the context of ALS, if indeed there is not only an association (Lagrange et al., 2021b) but an actual cause-effect relationship with ingestion of *Gyromitra* spp. Testing that relationship requires a detailed understanding of the lifetime exposome prior to onset of motor symptoms, not the rather superficial knowledge of exposures gained through epidemiological surveys of ALS, which have tended to emphasize occupational exposures (Holloway and Mitchell, 1986; Chancellor et al., 1993; Graham et al., 1997; GBD 2016 Motor Neuron Disease Collaborators, 2018) and have rarely examined dietary history. An example of studying individual subjects with uncommon forms of ALS is a 56-year-old man with a history of poisoning from eating either crude or undercooked false morels: he developed muscle cramps, nausea and vertigo, and a rapidly evolving/sub-acute upper and lower motor neuron syndrome with significant weakness in all four limbs and bulbar region; however, 6 months later, his condition plateaued, and he began a progressive recovery over subsequent years to reach normal neurological status with no electromyographic evidence of denervation (Lagrange et al., 2021a). Note that certain other *Gyromitra* spp. (*G. ambigua*, *G. infula*) are thought to contain gyromitrin (Brooks and Graeme, 2016), while other fungi contain related toxic azo/azoxy compounds (Andary et al., 1985; Flume, 2022), including the Stump Puffball [*Apioperdon* (formerly *Lycoperdon*) *pyriforme*] and Yellow Puffball (*Calvatia rubroflava*), which are considered to be edible (Blair and Sperry, 2013).

### ALS and non-botanical exposures to hydrazinic chemicals

There is potential for significant non-botanical exposures to hydrazine-related chemicals in industrial, agricultural and military settings. Their principal applications include chemical blowing agents (40%), agricultural pesticides (25%), and water treatment (20%) (Wiser, 2022). In the military and in aerospace, hydrazine has been used in various rocket fuels, in fuel cells to power an experimental Army truck (Anon, 2022g) and to fuel the EPUs of the NASA Space Shuttle, the Lockheed U-2 Spy Plane, and the General Dynamics F-16 fighter jet (Mayfield, 2022). Given America’s heavy use of F-16s in the 1991 Gulf War and the potential for hydrazine exposure of those who flew and serviced these aircraft, there is reason to explore a possible association with the high incidence of ALS among relatively young subjects who served in the U.S. Air Force at that time (Horner et al., 2008). The proposal that inhalation of aerosolized cyanotoxins (notably L-BMAA) in desert sands was a significant factor for the development of Gulf War-related ALS (Cox et al., 2009) cannot explain why the disease only developed in isolated members of the U.S. military. For reasons unknown, even during later periods (2002–2005), ALS continued to impact the U.S. Air Force more than other armed services (Sagiraju et al., 2020).

Some ALS researchers have identified agrochemicals, notably the broad class of pesticides, as plausible causal factors for sALS among farmers and other rural residents (Granieri et al., 1988; Poloni et al., 1997; Govoni et al., 2005; Gams et al., 2018; Povedano et al., 2018; de Jongh et al., 2021). Hydrazine compounds are used as active ingredients in combination with other agricultural chemicals, including insecticides, miticides, nematicides, fungicides, antiviral agents, attractants, herbicides, and plant growth regulators (Toki et al., 1994). Specifically, it is noteworthy that a synthetic hydrazine-related compound (maleic hydrazide: 1,2-dihydro-3,6-pyridazinedione), a plant-growth regulator synthesized in 1947 (Schoene and Hoffman, 1949) and introduced in the U.K. in 1984 (EFSA, 2016), served as a first-generation plant regulator for turf management in sports fields (Anon, 2015). Use of a hydrazine-related compound to control turf on sports fields has obvious potential relevance to the higher risk for ALS reported among relatively young professional and amateur soccer players (Vanacore et al., 2006; Chiò et al., 2009; Beghi, 2013). Factors relevant to this consideration include: the stability of maleic hydrazide in (simulated) sunlight, its slow degradation *in situ*, the lack of product odor, and the potential for exposure through inhalation and dermal contact (PubChem, 2022), and (b) the paucity of genetic fast acetylators in European (5–10%) as compared to Japanese (45%) populations (Koizumi et al., 1998). Hydrazine-related chemicals have also found use as herbicides (Metribuzin, Paclobutrazol) and fungicides (Triademifon) (Toki et al., 1994), and nitrosamines (*vide supra*) are released from recycled rubber crumb used in artificial turf (United States Environmental Protection, 2022; van Bruggen et al., 2022).

Occupational exposure to hydrazine and nitrosamines has occurred in the leather industry (de la Burde et al., 1963; Lahiri et al., 1988), which historically has had elevated rates of ALS (Hawkes and Fox, 1981; Buckley et al., 1983), a subject discussed elsewhere (Spencer, 2019), as well as an excess of stillbirths and an increased number of congenital malformations (Hawkes et al., 1989). Textile workers, who work with azo (hydrazone) dyes (Benkhaya et al., 2020) have also been identified as possible subjects at risk for ALS (Abarbanel et al., 1985, 1989). In the 1970s, azo dyes that form aromatic amines were used in hair rinses and tints (Ames et al., 1975; Yung and Richardson, 2004), which has potential relevance to the report of an elevated risk of ALS among hairdressers (Chió et al., 1991; D’Ovidio et al., 2017).

### ALS exposome research

Experience with migrants to and from Guam demonstrates that years or decades separate the timing of exposure to an environmental trigger (cycad genotoxins) and clinical onset of motor neuron disease. Additional experimental evidence indicates that exposure to the culpable agent (primarily cycasin) can occur prior to or after birth (Spencer, 2020), during infancy or in adolescence, although the latter may be a period of greatest vulnerability (Spencer et al., 2020). Detailed assessment of an ALS patient’s exposure to extrinsic factors from conception to onset (lifetime exposome) is a daunting task (Maguire, 2017). In general, one searches both for unusual high exposures to commonly encountered exogenous agents as well as exposures to agents to which few are in contact, whether the agent itself or the history of exposure deviates substantially from the norm. Such exposures, even to a single chemical class (such as hydrazine-related compounds), may involve a wealth of natural and synthetic substances deployed in multiple loci that cannot be addressed by conventional epidemiologic methods. Indeed, the “neural exposome,” a construct recently introduced by the NIH-National Institute of Neurological Disease and Stroke, comprises not only exogenous factors but also behavioral and endogenous components (Tamiz et al., 2022). When available, the science of exposomics may employ internal and external exposure assessment methods (Vasta et al., 2021). While research on the selected group of ALS cases highlighted here may be questioned, it is important to recognize that major discoveries of the causes of both acute and chronic neurologic diseases have been made by intensive study of very small numbers of patients. For example, end-stage L-dopa-responsive parkinsonism in a handful of post-teenage males was traced to drug use of a meperidine analog contaminated with methylphenyltetrahydropyridine (MPTP) (Langston, 2017).

Since onset of sALS in younger vs. older persons might plausibly result from a higher dosage of culpable agent(s) at a critical point in life, patients who are diagnosed with sALS in their second to fourth decade represent invaluable subjects for research investigation. Such cases are also more likely to have living parents and older siblings able to describe the ALS subject’s environmental exposures that occurred from conception onward. Our experience has taught that interviews with the patient and their family members are best conducted in a semi-structured manner (Palmer and Spencer, 2002) without rigid adherence to a research instrument with pre-formed questions that often reflect researcher interests or biases, such as the commonly perceived overarching etiologic relevance to sALS of workplace chemical exposure. The power of a non-biased approach to the acquisition of local knowledge was demonstrated by several Guam Chamorros who suggested six decades ago that food use of cycad was responsible for *leetiko* (ALS) in their community, an observation that led to the identification in cycad seed of glucosides of methylazoxymethanol (MAM) and the non-protein amino acid alpha-amino-beta-methylaminopropionic acid (Whiting, 1988), later named L-BMAA (Nunn, 2017). Over the course of six NIH-sponsored cycad conferences (1962–1972), discovery that MAM (like MMH) is a genotoxin with carcinogenic properties, prematurely diverted interest away from neurology and toward cancer biology. Since MAM exposures induce DNA damage in both cycling and non-cycling cells (i.e., neurons), this may result in tumorigenesis of the former and degeneration of the latter. Concurrent study of cancer and sALS may thus be merited in the search for genotoxic mechanisms as discussed elsewhere (Bharucha et al., 1983; Eizirik and Kisby, 1995; Spencer et al., 2012; Fang et al., 2013; Bertuzzo et al., 2015; Gibson et al., 2016; Taguchi and Wang, 2017).

Evidence from studies of familial ALS and sALS suggests that an interplay among DNA damage, altered DNA repair, and changes in epigenetic pattern, contribute to neurodegeneration (Goutman et al., 2022). Genome-wide DNA methylation age is the most consistently altered epigenetic signature in ALS. In twins with ALS, there was a much greater between-co-twin difference in DNA methylation age in a late-onset sALS twin set compared to an early-onset sALS twin set (Tarr et al., 2019). Noteworthy is that hydrazine induced hypomethylation of c-jun and p53, and hypermethylation of c-Ha-ras and DNA methyltransferase, in rodent liver (Kuppusamy et al., 2015). The experimental DNA-damaging activity of hydrazine-related compounds, including hydrazine hydrate and 1,2-methylhydrazine (which is metabolized to MAM), is well established in the cancer literature (Pollard and Zedeck, 1978; Parodi et al., 1981), and experimental systemic treatment of young adult mice with MAM induces brain transcriptional profiles associated with both cancer and neurodegenerative disease (Kisby et al., 2011).

### Future research direction

We propose there are special opportunities for discovery of environmental factors associated with/causal of ALS from intensive analysis of the lifetime exposome of patients with young-onset sporadic disease, as well as very rare ALS-discordant twin and conjugal ALS cases (Spencer et al., 2019, 2022a). Since the lifetime probability of conjugal ALS (in Britain) has been estimated as 1/510,000 couples (∼0.75 couples/year) (Cornblath et al., 1993; Fernandes et al., 2017), less rare, young onset sALS cases (45 years of age and younger) represent priority research subjects. While sALS occasionally occurs in the second decade of life, such cases should also be screened for mutant genes since juvenile-onset genetic forms of ALS occur in children and subjects < 25 years of age (Turner et al., 2012). Genetic screening, exposome analysis and the timing of exposure may also reveal evidence of postulated gene-environment interactions in ALS (Bradley et al., 2018; Goutman et al., 2022). Environmental exposures should cover both synthetic and natural agents, including mycotoxins, given their potential relevance to the etiology of ALS (Alonso et al., 2015; Reid, 2017, 2020; French et al., 2019a,b).

Retrospective analysis of sALS clusters and geographically proximate cases is another valuable research strategy. This approach requires collaboration among ALS neurologists, epidemiologists, mycologists, analytical chemists, toxicologists, and other specialists, often with input from members of ALS-affected communities. Given the recent recognition of an association between sALS and prior False Morel poisoning in the French Alps (Lagrange et al., 2021b), an association that should be tested in other populations, prospective research strategies should include long-term follow-up of patients with a history of acute MMH poisoning identified by poison control centers. While the focus here is on the environmental etiology of ALS and its potential relationship to hydrazine-related chemicals, the hypothesis may be pertinent to other neurological disorders, since cycad-associated Western Pacific ALS phenotypes included atypical parkinsonism and progressive Alzheimer-like dementia (Borenstein et al., 2007; Spencer et al., 2020).

## Author contributions

PS drafted the manuscript. VP provided the Gulf War research data. GK contributed to genotoxic data. EL, J-PV, CR, JR, and WC provided the data on ALS in the French Alps. RV contributed to data on mushroom occurrence in Spain. BH provided the data on U.S. cases of monomethylhydrazine poisoning. All authors edited and/or read the manuscript.

## Footnotes

1
https://vimeo.com/1621281

2
https://www.pbs.org/video/als-xmw7rw/

3
https://en-academic.com/dic.nsf/enwiki/182112
